# Aesthetic preferences of the nasolabial angle: A three-dimensional (3D) study

**DOI:** 10.1016/j.jpra.2025.08.026

**Published:** 2025-08-28

**Authors:** S. El-Habbash, A. Bakshi, B.S. Khambay

**Affiliations:** a Postgraduate Student, Institute of Clinical Sciences College of Medical and Dental Sciences, The School of Dentistry, University of Birmingham, 5 Mill Pool Way, Edgbaston, Birmingham, B5 7EG, United Kingdom; b Institute of Clinical Sciences, College of Medical & Dental Sciences, The School of Dentistry, University of Birmingham, 5 Mill Pool Way, Edgbaston, Birmingham, B5 7EG, United Kingdom

**Keywords:** Nasolabial angle, Perception, Attractiveness, Three-dimensional

## Abstract

Historically, the ideal nasolabial angle (NLA) ranges from 90° to 120°. This study was conducted to evaluate the current preferences of NLA of laypeople and clinicians based on 3D female textured and 3D male textured images. Three-dimensional software (Di3DView) was used to create a 3D facial image with a NLA of 70°. The colour images of one male and female adult were remapped onto the generated 3D image, resulting in a colour textured 3D male, and 3D female image, both with the same facial shape and a 70° NLA. This process was repeated for NLA’s of 80°, 90°, 100°, and 110°. Based on a 7-point Likert scale, seventy-two laypeople and fifty clinicians were asked to evaluate the NLA of each rotating 3D image and give a facial attractiveness score. Clinicians and laypeople showed good intra-rater reliability (ICC 0.75 to 0.89). A 90° NLA was found to be most attractive for 3D male textured images (*p*=0.001). Whilst for 3D female textured images, an 80° and 90° NLA was found to be the most attractive (*p*=0.001). Laypeople were more critical than clinicians. Both groups would seek treatment for NLA of 70°, 100°, and 110°. We were able to show that 90° NLA’s were preferred for males and 80°–90° NLA’s for females. Laypeople were more critical of obtuse nasolabial angles, and less critical of acute nasolabial angles than clinicians. Obtuse NLA’s angles greater than 90° are no longer considered acceptable.

## Introduction

The nasolabial region plays a pivotal role in facial aesthetics.^1^ Achieving a normal nasolabial angle (NLA) contributes to restoring facial balance and facilitating the attainment of aesthetic objectives.^2^ Historical values for an ideal NLA, range between 90° and 120°.^3^ The range of acceptance of the NLA is influenced by variables such as gender and ethnicity.^4^ Cultural and ethnic differences significantly shape aesthetic preferences, with more acute NLA favoured in African populations compared to Caucasian and Asian populations.^5^ The NLA can alter as a result aging, orthodontic treatment, orthognathic surgery or a rhinoplasty.^6^^,^^7^ Similarly, following a rhinoplasty, nasal projection, nasal tip position and rotation can all significantly impact the NLA.^8^

The evolution of societal beauty standards, driven by increased exposure to social media and advancements in cosmetic treatments has meant that more acute NLA’s are now perceived as more attractive.^9^ Consequently, it is important for orthodontists and surgeons to consider treatment objectives in accordance with patient’s expectations and perceptions relative to prevailing standards. This necessitates a consensus on NLA norms among both clinicians and laypeople, acknowledging changes in perception over time.

Previous studies have either assessed nasolabial preference based on two-dimensional (2D) profile silhouettes, modified colour 2D profile photographs of individuals, or 2D profile photographs of groups of individuals with specific nasolabial angles.^10^, ^11^, ^12^ Each of these approaches has its own limitations; 2D silhouette profiles lack texture, are neither male or female, and are not routinely viewed by clinicians or laypeople. Modified colour 2D facial profile images are also 2D, are susceptible to magnification errors and distortions, and are unable to capture depth and three-dimensional facial contours.^13^^,^^14^ Finally, comparing groups of individuals with different nasolabial angles introduces additional confounding variables, such as skin complexion and hair style, which could potentially influence the rater’s perception of attractiveness.

The use of 3D facial images has been shown to be crucial when assessing facial attractiveness and reducing potential biases by enabling the evaluation of profiles from multiple angles.^15^ It is surprising that there are only a limited number of studies utilising 3D imaging to assess patient perceptions using modified male and female textured images.^16^ Three-dimensional facial images contain two separate types of information, the surface geometry of the face and the photorealistic colour facial texture information. It is possible to maintain surface geometry and change the overlying colour texture information. This means, a facial mesh with a 90° NLA can be generated (surface geometry) and overlaid with either a male or female photorealistic colour facial texture. A rater viewing these two images, will be viewing a face with the same NLA and surface geometry, but either as a male or female. This methodology addresses several of the shortcomings of the previously discussed image types.

The aim of this study was to determine which nasolabial angle (ranging from 70° to 110°) was scored as most attractive using 3D female and 3D male textured images. The primary outcome measure was the difference in facial attractiveness score. The Null hypothesis was that there was no statistically significant difference (*p*<0.05) in facial attractiveness rating score between the 5 different nasolabial angle (70°, 80°, 90°, 100°, and 110°). The secondary outcome were differences in facial attractiveness scores between laypeople and clinicians. Additionally, the study aimed to identify the nasolabial angles that would prompt clinicians and laypeople to seek treatment.

## Materials and methods

### Ethical approval

Ethical approval for this study was sought and granted by the University of Birmingham’s research and ethics committee (ERN 20-1332). Verbal and written informed consent had been obtained. All participants gave written informed consent.

### Sample size calculation

A priori sample size calculation was conducted using G*Power.^17^ Parameters for the calculation included a power of 90 % (0.9), a statistical significance level of *p* = 0.05, and an effect size of 0.2.^18^ An additional 15 % was added to the calculated sample size to account for incomplete data entry. This produced a minimum requirement of 46 raters in each group: clinicians and laypeople.

### Three-dimensional image generation

#### Male and female texture capture

Two Caucasian adult volunteers, one clean-shaven male and one female, were imaged in rest position. Both were staff members at Birmingham Dental Hospital and School and had no facial scars and had good skin complexion. The 3D facial images were captured using the Di4DSNAP imaging system (Dimensional Imaging Ltd, Hillington, Glasgow). This system comprised six digital cameras arranged in three banks of two, connected to a personal computer. Calibration of the system was performed as per the manufacturer’s instructions before image capture. The facial images were saved as wavefront (.OBJ) files, containing the surface geometry and texture coordinates. The texture coordinates were used to project the 2D image onto the 3D model's surface for texture mapping. These files were accompanied by a material library (.MTL) file describing surface shading properties, and the 2D texture image file (.JPEG or .BMP).

#### Transfer of male and female colour texture

**Figure 1:**
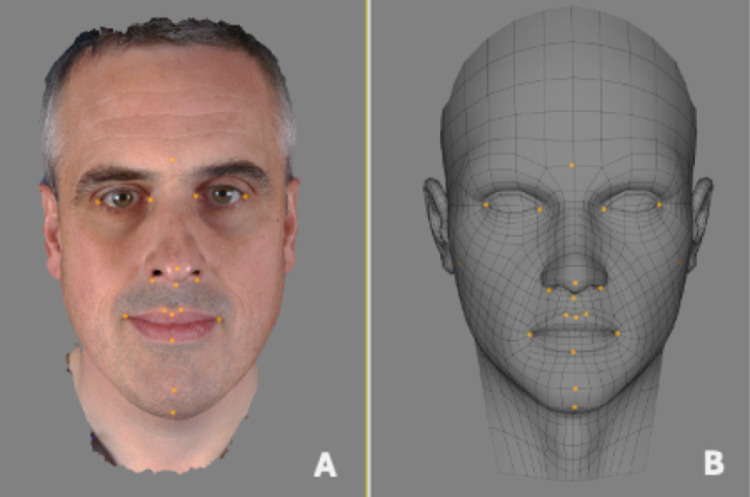
Twenty-two corresponding anatomical landmarks placed on original image (A) used to elastically deform the generic mesh (B).

**Figure 2:**
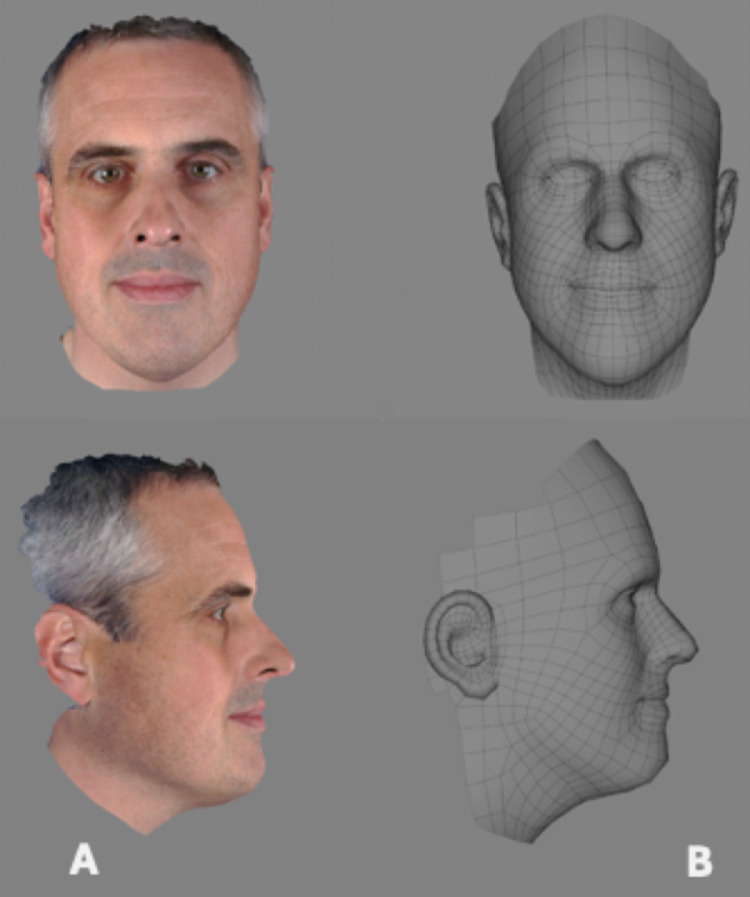
Following the 'Shape Transfer' function in Di3DView the generic mesh (A) was elastically deformed to the original topography (B).

**Figure 3:**
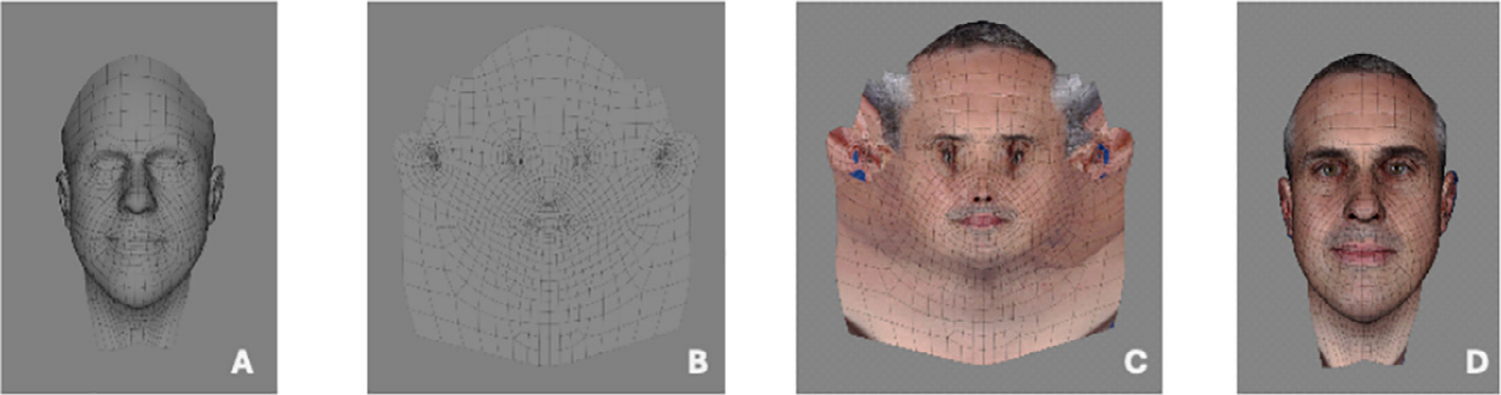
Conformed generic mesh (A) unwrapped (B). 2D photograph re-mapped to the new conformed generic mesh (C) and re-wrapped to a 3D image (D).

Using Di3DView software, the original wireframe mesh (surface geometry), for the male 3D image, was replaced with a 'generic mesh', a symmetrical, textureless 3D facial mesh, Figure 1. Using the 'shape transfer' function the generic mesh was elastically deformed to match the original wireframe’s topography, constrained on 22 anatomical landmarks, Figure 2. The male 2D texture image was then remapped to the conformed mesh using the 'Material Transfer' function, and a new 3D facial image produced, Figure 3. The output was saved as a new .OBJ file, made up of the conformed generic mesh and the original male texture file (.JPEG). The same procedure was repeated for the female subject, resulting in another .OBJ file made up of the conformed generic mesh and the original female texture file (.JPEG). As the two conformed generic meshes, male and female, were based on the same generic mesh the texture coordinates were identical. This allowed the male and female textures to be both mapped to the same generic mesh.

#### Modification of nasolabial angles

**Figure 4:**
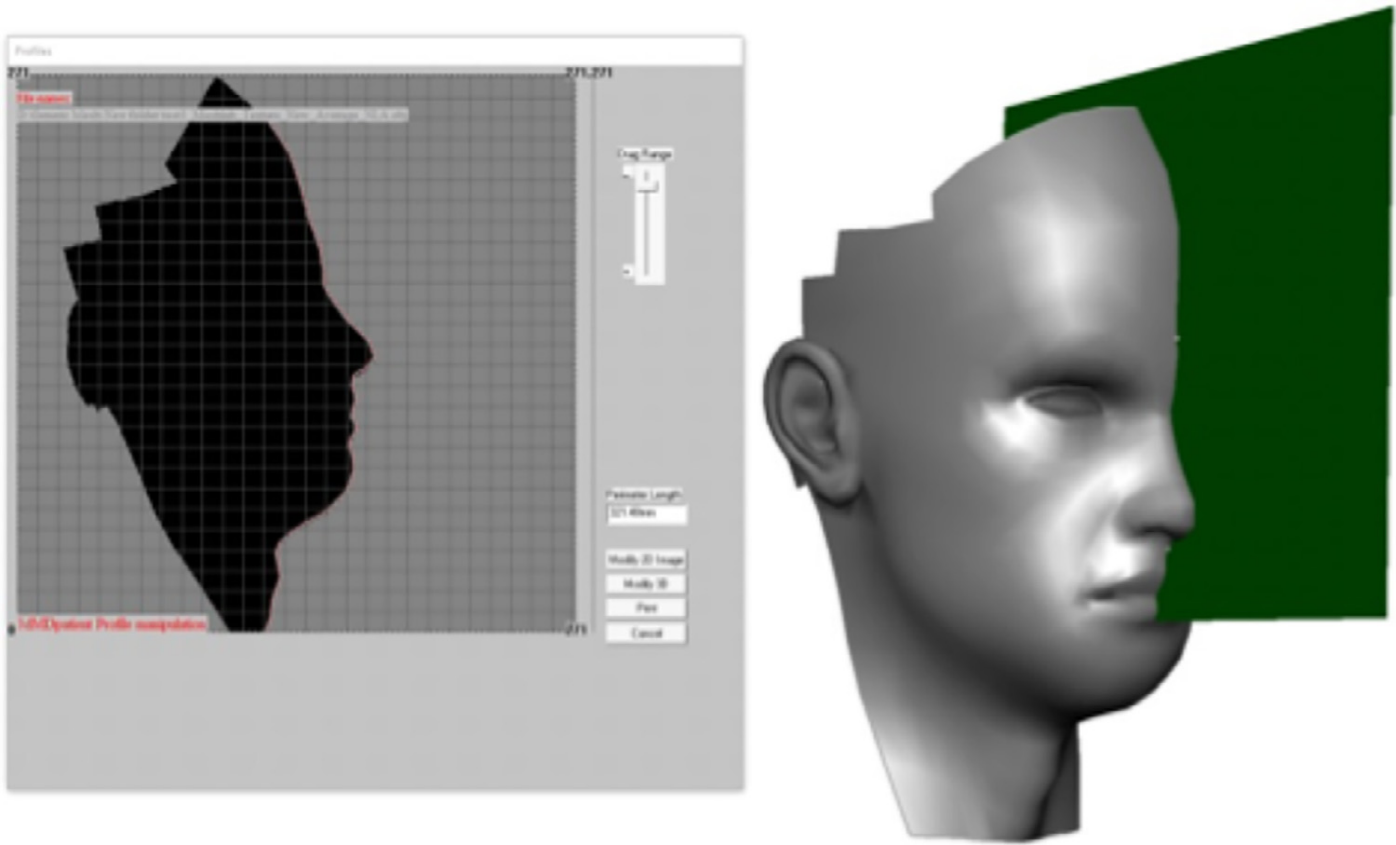
The use of 3dMD Patient software to adjust the 2D profile image generated by the sagittal plane (green).

The generic mesh was imported into 3dMD Patient software (Atlanta, Ga) to adjust the nasolabial angles. A facial mid-plane was created by manually identifying three anatomical points: Glabella, Nasal Tip, and Subnasale. Using the 'Free-form' tool, the nasolabial angle was modified to values of 70°, 80°, 90°, 100°, and 110°, with changes reflected in the 3D surface, Figure 4. Each modified model was saved as a separate .OBJ file.

#### Addition of texture to modified 3D facial image – male and female

**Figure 5:**
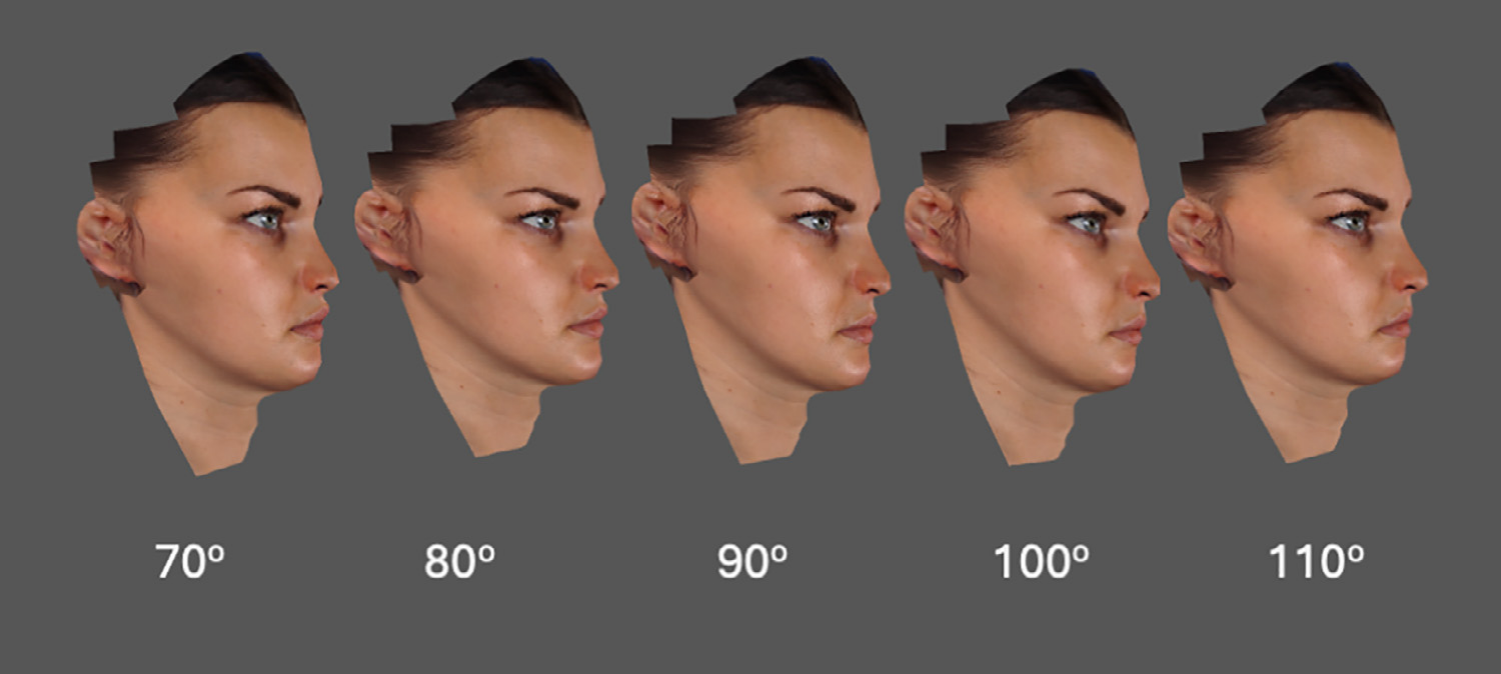
Female textured image (.JPEG) wrapped over each conformed generic mesh with nasolabial angles modified to values of 70°, 80°, 90°, 100°, and 110°.

**Figure 6:**
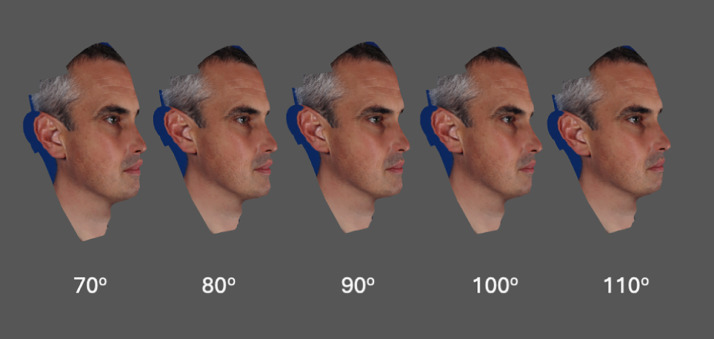
Male textured image (.JPEG) wrapped over each conformed generic mesh with nasolabial angles modified to values of 70°, 80°, 90°, 100°, and 110°.

As highlighted previously the conformed generic meshes for both the male and female subject were constructed from the same generic mesh and texture coordinates. Therefore, either the male or female textured image (.JPEG) could be used with the generic mesh. In other words, the female texture map could be loaded over the male facial surface shape and vice versa, Figs. 5 and 6.

### Preparation of visual stimuli

#### PowerPoint embedding

Using screen recording software (Screenrec, San Francisco, United States), each 3D image was recorded, and saved in .MP4 format, rotating from the left ear to the right ear over a 30 s duration. For ease and reproducibility of viewing, the video sequences (.MP4) were embedded into a PowerPoint presentation. Images were grouped by category 3D textured (male) and 3D textured (female). Within each group the different nasolabial angle images appeared in a random order. In addition, six duplicate images, three from each gender, were randomly selected and included for reliability testing purposes. In total, 16 images / videos were produced for viewing.

### Rater recruitment and procedure

#### Clinician group

Fifty clinicians, including registered specialist orthodontists and trainees, were recruited through the West Midlands Orthodontic Society, Northern Universities Consortium, and Birmingham Dental Hospital. Inclusion criteria included orthodontic registration and experience as a specialist or trainee. Data collection occurred virtually via Microsoft Teams. Participants completed either paper-based or electronic scoring sheets.

#### Laypeople group

Seventy-two laypeople were recruited from the University of Birmingham’s School of Medical Science. Inclusion criteria included adults aged over 18 with no history of facial surgery, deformities, or trauma. Data collection occurred virtually via Microsoft Teams. Participants completed either paper-based or electronic scoring sheets.

Participants viewed each image for 30 s during the rating presentation. Once completed, two separate EXCEL sheets were created, one for the clinician group and the other for the laypeople group. The Excel sheets were completed with coded information to allow the anonymous data to be analysed.

### Rating procedure

Participants rated images using a 7-point Likert scale, assessing the nasolabial region. The scale ranged from one (extremely unattractive) to seven (extremely attractive). Written and verbal instructions were provided before rating began, with raters being given the opportunity to ask any questions prior to rating any images. Raters also answered the follow-up question, ‘Would you consider seeking treatment if this image represented your own appearance?’ (Yes or No).

The data was tested for normality and found not to be normally distributed. Therefore median and interquartile ranges were reported. Intra-rater reliability was assessed using the absolute agreement intraclass correlation coefficients (ICC), two-way fixed effects model and single measurement.

A Friedman test was performed to determine if there were differences in facial attractiveness scores, between the five different nasolabial angles when viewing 3D female textured and 3D male textured within the laypeople group and the clinician group. Pairwise comparisons were performed (SPSS Statistics, 2012) with a Bonferroni correction for multiple comparisons.

A Mann-Whitney U test was utilised to determine if there were statistically significant differences (*p*=0.05) in attractiveness ratings between clinicians and laypeople across the same five nasolabial angles.

## Results

### Rater demographics

The clinician and laypeople raters age, gender and race are shown in Table 1.

**Table 1:** Demographics of clinician and laypeople groups.

Age		
Age (years)	Clinician	Laypeople
18 - 44	42	72
45 - 65	8	0
Gender		
Gender	Clinician	Laypeople
Female	25	23
Male	25	49
Race		
Race	Clinician	Laypeople
Bangladeshi	0	6
Indian	13	26
Pakistani	3	13
Any other Asian background	4	4
African	0	1
Any other black background	0	1
Black and white African	1	0
Asian and White	1	0
Any other mixed background	0	4
British	12	9
English	2	2
Irish	3	0
Welsh	3	2
Any other white background	0	1
Chinese	1	0
Arab	5	1
Any other	1	1
Prefer not to say	0	1

### Intrarater reliability

For both the clinicians and laypeople the ICC was between 0.80 and 0.86 for clinicians and 0.75 to 0.89, indicating a good level of reliability.

### 3D male and 3D female textured images

#### Different nasolabial angles

For both male and female 3D image types, following a Friedman test, the median facial attractiveness scores were statistically significantly different between the five different nasolabial angles (*p*<0.001), for both the clinician and laypeople groups. Following a three-way ANOVA there was no statistically significant three-way interaction between gender, age and clinical grade on facial attractiveness score in the clinician group. In the laypeople group, following a two-way ANOVA there was no statistically significant two-way interaction between gender and age on facial attractiveness score. The percentage of laypeople and clinicians that found the NLA attractive (Likert scale 5 to 7 inclusive) for 3D female and male textured images is shown in Table 2.

**Table 2:** Percentage of laypeople and clinicians that found each nasolabial angle attractive (Likert scale 5 to 7 inclusive) for 3D female and male textured images.

Viewing image	Clinicians					Laypersons				
	Nasolabial angle					Nasolabial angle				
	70°	80°	90°	100°	110°	70°	80°	90°	100°	110°
3D female textured	18.8 %	64.6 %	64.6 %	4.2 %	2.1 %	31.9 %	58.3 %	31.9 %	6.9 %	4.2 %
3D male textured	0.0 %	14.6 %	93.8 %	14.6 %	18.8 %	6.9 %	30.6 %	54.2 %	5.6 %	4.2 %

### 3D male textured images

#### Different nasolabial angles

Post hoc analysis showed that the male textured image with a 90° nasolabial angle was found to be the most attractive (*p*=0.001), compared to 70°, 80°, 100° and 110° nasolabial angles, by both the clinician and laypeople groups.

#### Laypeople versus clinician group

The laypeople group reported that the 90° nasolabial angle was less attractive (*p*=0.001), Table 3. In addition, the laypeople group found that males with 100° and 110° nasolabial angles were less attractive (*p*=0.001) and that males with 70° nasolabial angles were more attractive (*p*=0.022), compared to the clinician group.

**Table 3:** Median facial attractiveness scores for the laypeople and clinician group assessing five different nasolabial angles.

	Layperson group		Clinician group		
Nasolabial angle	Median score	IQR	Median score	IQR	p-value
3D male textured image					
70 ^o^	3	3 to 4	3	2 to 3	0.022*
80 ^o^	4	3 to 5	4	3 to 4	0.217
90 ^o^	5	4 to 5	6	5 to 6	0.001*
100 ^o^	3	2 to 3	3	3 to 4	0.001*
110 ^o^	3	2 to 3	3.5	3 to 4	0.001*
3D female textured image					
70 ^o^	4	3 to 5	3	3 to 4	0.171
80 ^o^	5	4 to 5	5	4 to 6	0.036*
90 ^o^	4	3 to 5	5	4 to 6	0.001*
100 ^o^	3	2 to 3	3	2 to 3	0.713
110 ^o^	2	2 to 3	2	2 to 3	0.944

Difference in attractiveness rating between the clinician and laypeople group following a Mann-Whitney U test *(*p*<0.05).

1-Extremely unattractive.

2-Very unattractive.

3-Slightly unattractive.

4-Neither attractive nor unattractive.

5-Slightly attractive.

6-Very attractive.

7-Extremely attractive.

### 3D female textured images

#### Different nasolabial angles

3D female textured images with an 80° and 90° nasolabial angle were found to be the most attractive (*p*=0.001), by both the clinician and laypeople groups, compared to 70°, 100° and 110° nasolabial angles.

#### Laypeople versus clinician group

The laypeople group found that females with 80° and 90° nasolabial angles were less attractive than the clinician group (*p*=0.036 and *p*=0.001 respectively), Table 3. Both clinicians and laypeople gave similar facial attractiveness score for 70°, 100° and 110° nasolabial angles.

### Nasolabial angle and seeking treatment

**Figure 7:**
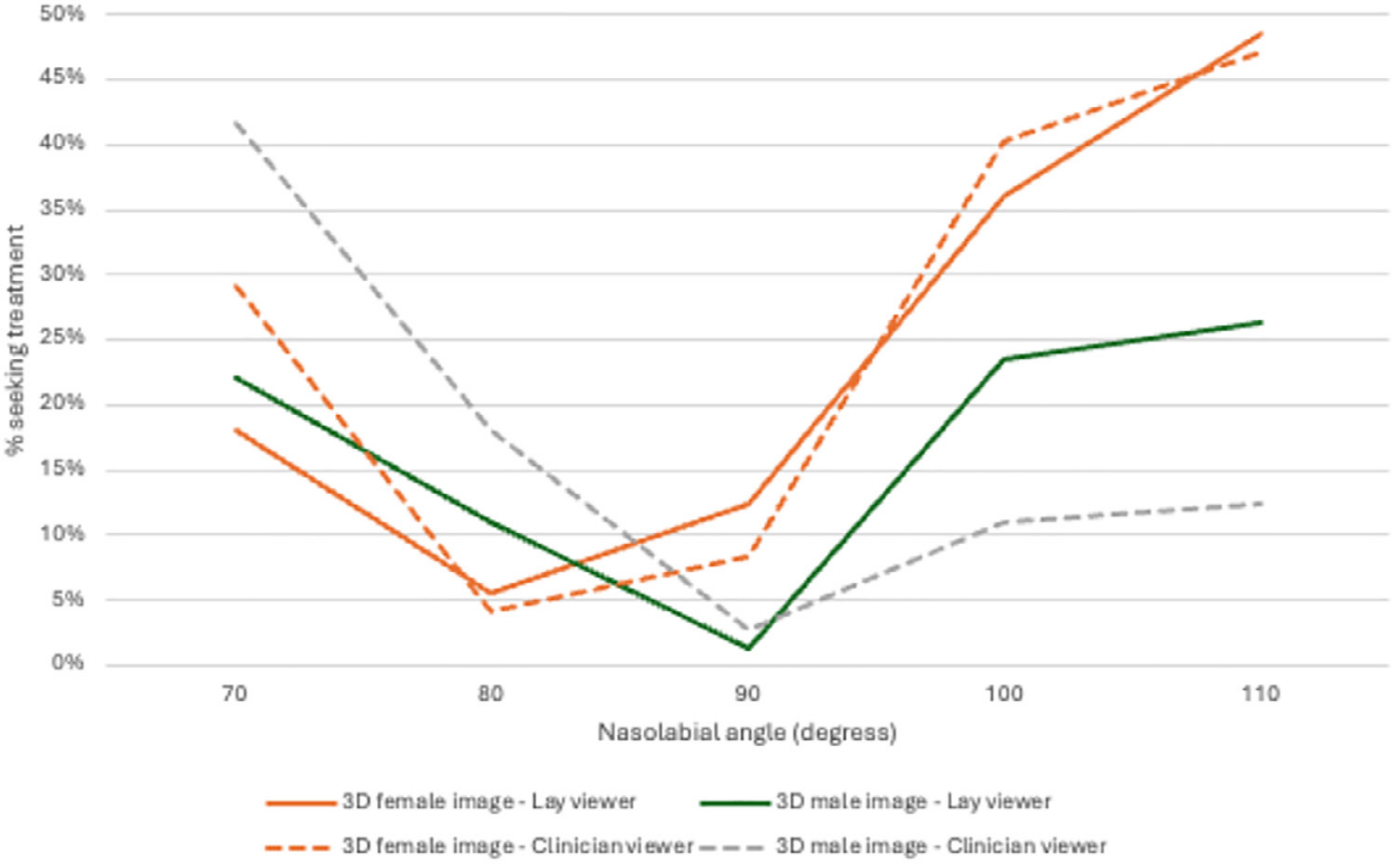
Shows the percentage of clinicians (Clinician) and laypeople (Lay) seeking treatment 3D female texture image and the 3D male textured image.

The percentage of clinicians and laypeople seeking treatment for each nasolabial angle is shown in Table 4 and Figure 7.

**Table 4:** The percentage of clinicians and laypeople who would seek treatment for each nasolabial angle, along with the viewing media that influenced their decision.

Viewing image	Clinicians					Laypersons				
	Nasolabial angle					Nasolabial angle				
	70°	80°	90°	100°	110°	70°	80°	90°	100°	110°
3D female textured	29.2 %	4.2 %	8.3 %	40.3 %	47.2 %	18.1 %	5.6 %	12.5 %	36.1 %	48.6 %
3D male textured	41.7 %	18.1 %	2.8 %	11.1 %	12.5 %	22.2 %	11.1 %	1.4 %	23.6 %	26.4 %

### Three-dimensional (3D) female textured images

The highest percentage of clinicians and laypeople seeking treatment were for nasolabial angles of 110° and 100° angles. Only 4.2 % of clinicians and 5.6 % of laypeople would seek treatment for an 80° nasolabial angle.

### Three-dimensional (3D) male textured images

The percentage of laypeople seeking treatment were for nasolabial angles of 70°, 110° and 100° angles, were similar. However 41.7 % of the clinician group would seek treatment for a 70° nasolabial angle and half the number of clinicians for 110° and 100° nasolabial angles.

## Discussion

Historically, normative population values for nasolabial angle have originated from various sources, including anecdotal data, normative cephalometeric studies, profile photographs measurements and from rating profile silhouettes.^3^^,^^10^, ^11^, ^12^ Modified profile silhouette images have been previously rated by clinicians, patients and laypeople, but laypeople and patients will have had little exposure to profile silhouettes and may find it difficult to interpret the images from a clinical perspective.^13^, ^14^ Humans recognise caricatures and silhouettes of images by firstly scanning for general patterns in an image and then, if time permits, acquiring more detailed information. This is believed to be more efficient as it allows rapid assessment of the visual situation and time to respond appropriately. Unfortunately, this may lead to erroneous outcomes.^19^ In addition, the human eye is more sensitive to colour than to black and white images as well as depth. The use of three-dimensional images should address some of the shortcomings of profile silhouettes as they contain texture, anatomical detail and depth.^13^, ^14^, ^15^^,^^20^ The novelty of the present study was not only the use of standardised 3D facial images, with five different nasolabial angles, but the ability to add a male or female texture onto the 3D image. In addition, the 3D images were viewed as rotating images creating the perception of depth. This enabled direct comparison of the effect of gender on perceived nasolabial region facial attractiveness, whilst minimising confounding factors, such as differences in hair colour and skin complexion.

The results of the present study showed both clinicians and laypeople consistently rated 70°, 100° and 110° nasolabial angles as least attractive for both 3D female textured and 3D male textured images. In addition there was a general trend for laypeople to be more critical than clinicians. From this study, both laypeople and clinicians, rated a nasolabial angle of 80° - 90° for 3D female textured images and a nasolabial angle of 90° for 3D male textured images as the most attractive. These findings were not in total agreement with a recent scoping review on perceived attractiveness of nasolabial angle which reported a nasolabial angle range of 86° to 107° for men, and 84° to 123° for women was attractive.^5^ The review included 21 studies which were based on different raters (orthodontists, laypeople, and patients), different viewing media (colour photographs, black and white photographs, cephalograms, tracings, silhouettes and sketches) and different ethnic group subjects (African American, White, Japanese, Iranian and Persian). This heterogeneity may explain the wide range of nasolabial angles which were regarded as acceptable. Previous values for 'normal nasolabial angles' should be viewed with caution as they are either based on anecdotal / historical data or derived from measurements taken from individuals with attractive facial profiles. The fact that individuals have attractive facial profiles does not necessary mean that their nasolabial angle are ideal. Facial attractiveness is made up of many different attributes including youthfulness, symmetry, averageness and sexual dimorphism.^21^ So when raters are asked to score facial attractiveness, they are looking at the many different facial attributes and not specifically the nasolabial angle.

Secular facial profile changes have recently been reported in the literature and are significantly more noticeable in women’s profiles.^5^^,^^22^ This would imply the present historical normative database that drive clinical treatments, may no longer be valid in the 21st century. Presently and in the era of social media, the ideal of fuller lips with a more acute nasolabial angle in females has been reported.^5^^,^^23^ A cross-sectional study found that the nasolabial angle differed from historical studies, up to 40° more acute than previous studies.^23^ With a rise in lip fillers, which are becoming ever more popular, the impact on the nasolabial angle must be assessed and communicated with patients particularly if orthodontic or orthognathic treatment is undertaken.^24^

Historically, studies have found no association between the gender of the rater and facial attractiveness rating score.^25^ The results of the present study supported this finding and found there were no statistical difference in the facial attractiveness score between male and female clinicians or laypeople. In addition there was no association between age or degree of experience and facial attractiveness score in the clinician group. Many studies found no difference between gender and aesthetic perception.^26^, ^27^, ^28^

For 3D female textured images with nasolabial angles of 100° and 110° both clinicians and laypeople would have sought treatment. Whilst for 3D male textured images more laypeople would seek treatment than clinicians. Nevertheless, it is crucial to exercise caution when interpreting these results, as the laypeople group had a higher proportion of male participants, potentially leading to bias. Interestingly for both male and female images, laypeople were less likely to seek treatment with 70° nasolabial angles then the clinician group. This is an important finding as it suggests there is marked difference in perception between clinicians and laypeople for more acute nasolabial angles. This emphasises the importance of engaging with the patient and understanding their wishes and concerns and not treating individuals to a standard, or clinician preferences, particularly when invasive treatment such as orthognathic surgery is being considered.

There are several limitations with the current study, including the clinician group being made up of individuals of varying clinical experience including orthodontic speciality registrars in training, specialist orthodontists and consultant orthodontists. This study was carried out using 3D male and female textured Caucasian images only. Therefore, this study is not generalisable to other ethnic groups in terms of perceived aesthetics of the nasolabial angle as we know from previous studies that racial norms vary between ethnicities.^29^ The images in this study included male and female gender 3D textured images. However, in a changing age with gender neutrality and not identifying as either male or female, this may not be fully relevant to all groups of patients presenting for treatment.

## Conclusions

This study demonstrated that a wide range of NLA’s, between 90° and 120°, is no longer acceptable to clinicians and laypeople. 3D male textured images with 90° NLA’s were perceived as the most attractive, by both viewer groups. Layperson were more critical of obtuse nasolabial angles, and less critical of acute nasolabial angles, than clinicians. 3D female textured images with 80° and 90° NLA’s were perceived as the most attractive, by both viewer groups. 3D male and female textured images with nasolabial angles of 70°, 100°, and 110° were perceived as the least attractive by both raters, with both groups indicating a desire for treatment.

## Data availability

The data underlying this article will be shared on reasonable request to the corresponding author.

## Ethical approval

Ethical approval for this study was sought and granted by the University of Birmingham’s Research and Ethics Committee (ERN 20-1332).

## Declaration of Competing Interest

None.
